# Gastrointestinal Stromal Tumor: May Mimic Adnexal Mass

**DOI:** 10.5539/gjhs.v8n2p20

**Published:** 2015-06-01

**Authors:** Nilay Karaca, Yaşam Kemal Akpak, Zeynep Tatar, Gonca Batmaz, Aslihan Erken

**Affiliations:** 1 Bezmi Alem Vakif University, Medical Faculty, Department of Obstetrics and Gynecology, Istanbul, Turkey; 2 Ankara Mevki Military Hospital, Department of Obstetrics and Gynecology, Ankara, Turkey; 3 Patomer Pathology Center, Istanbul, Turkey; 4 TDV 29 Mayis Hospital, Department of Assisted Reproduction, Istanbul, Turkey

**Keywords:** preoperative diagnosis, gastrointestinal stromal tumor, GISTs, Adnexal mass, C-KIT, surgical treatment, tyrosine kinase

## Abstract

Gastrointestinal stromal tumors (GISTs) are rare tumor of the gastrointestinal tract. GISTs occur in the entire gastrointestinal tract and may also arise from the retroperitoneum, omentum and mesenteries. They are originated from gastrointestinal pacemaker cells (Cajal’s interstitial cells) and range from benign tumors to sarcomas at all sites of occurrence. Diagnosis of GIST could be deceptive because of their similarity in appearance to gynecological neoplasms. We would like to present a case of a woman with GIST in the small intestine giving a imprint of an adnexal mass was diagnosed correctly during surgery. The diagnosis and treatment of GIST has been reformed over the past years. It is crucial to separate GISTs from possible misdiagnosis because their prognosis and treatment could be unlike clearly. The purpose of this case is to evaluate this rarely seen clinical entity, and thus, make some contribution to the literature.

## 1. Introduction

Gastrointestinal stromal tumors (GIST) stemming from interstitial cells of Cajal known also as intestinal pacemaker cells are the most frequent mesenchymal tumors but they comprise <1% of all gastrointestinal tumors (Sarlomo-Rikala, Kovatich, Barusevicius, & Miettinen, 1998). Its incidence is estimated as 10-20 patients in 1 million population (P. Gupta, Tewari, & Shukla, 2008). The majority of patients are 40-80 years old. Mean age at diagnosis is almost 60 years. There is a slight male predominance in incidence (Pierie et al., 2001; Miettinen, Majidi, & Lasora, 2002). 60% of GISTs start in the stomach, 30% in the small intestine, 5-10% in colon and rectum and 5% in esophagus (Boldorini et al., 2001). Rarely, GIST may occur in areas outside the GI tract such as uterus, rectovaginal septum, vagina, mesentery and retro peritoneum (Van der Zwan & De Matteo, 2005). Clinical presentation of GISTs may vary and symptoms are related to their localization. The patients usually seek medical help for bleeding due to mucosal ulceration. Abdominal mass, nausea, vomiting, weight loss, abdominal discomfort and pain are among other symptoms (D’Amato, Steinert, Mc Auliffe, & Trent, 2005; Levy, Remotti, Thompson, Sobin, & Miettinen, 2003). Intraperitoneal GISTs may be detected as palpated abdominal mass, but occasionally they may be confused with ovarian mass (P. Gupta et al., 2008). Tyrosine kinase, CD34 and CD 117 receptor positivity can be used in differentiating GISTs from other mesenchymal masses (Van der Zwan & De Matteo, 2005).

Pre-operative diagnosis is difficult with this clinical picture and particularly in female patients GISTs may get confused with gynecological malignancies due to their location and symptoms. In this case report, with the guidance of literature we aim to discuss in details a patient whose suspected diagnosis was postmenopausal ovarian malignancy and operated accordingly but appeared to be a case of small intestine GIST during the operation.

## 2. Case Report

A 52 years old postmenopausal female patient has referred to our gynecology and obstetrics outpatient clinics in our hospital for routine gynecological exam. She was hypertensive for 5 years and had diabetes mellitus for 18 years. Gravida 3, para 2, abortus 1 patient had total vaginal hysterectomy operation 3 years ago because of abnormal uterine bleeding. Otherwise, she had no peculiarity in her medical history. Her general condition was good; BP 120/80 mmHg, heart rate 86/min and physical exam normal. In her gynecological exam vulva, vagina and vaginal cuff were normal in appearance; in bimanual exam a mobile mass app. 30-40 mm in size concordant with left adnexal area was detected.

**Figure 1:**
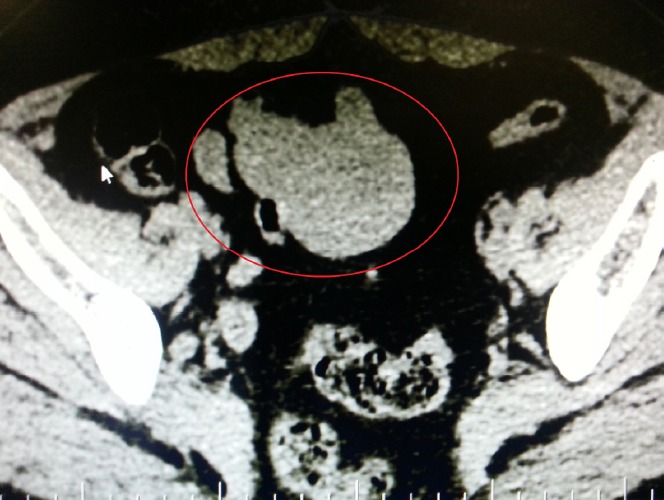
Bilobular appearing solid lesion 66×45 mm in size showing prominent contrast material fixation in enhanced images with a necrotic component in the centre

**Figure 2:**
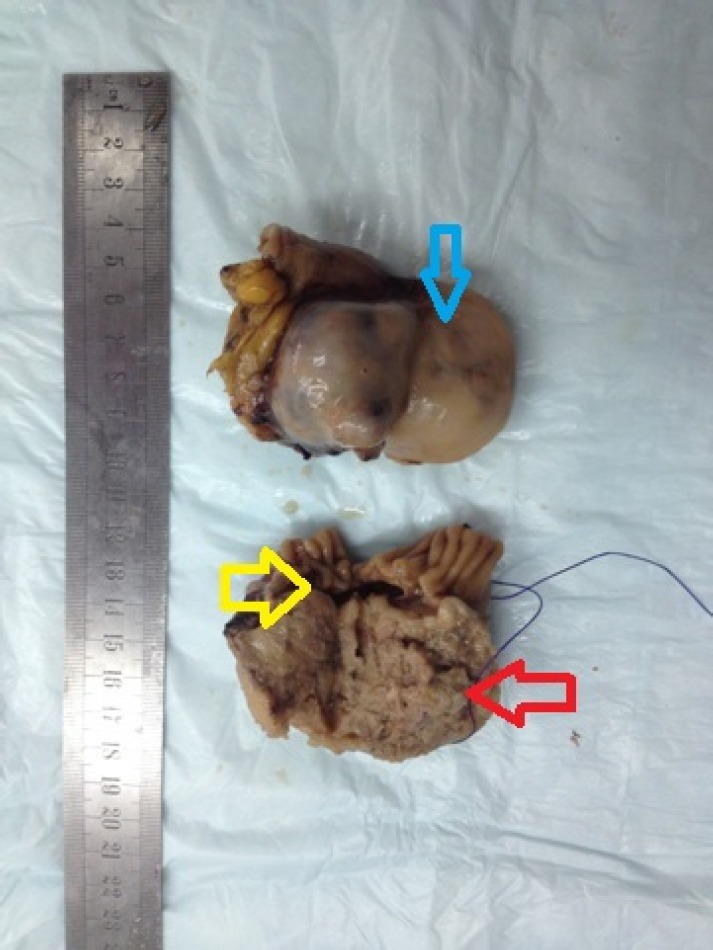
Tumoral mass with nodular features but with smooth surface 70×50×40 mm in size (blue and red arrows) adjacent to intestinal segment (yellow arrow). Cross sectional surface is relatively soft in consistency and includes occasional yellow areas (red arrow) concordant with necrosis

**Figure 3:**
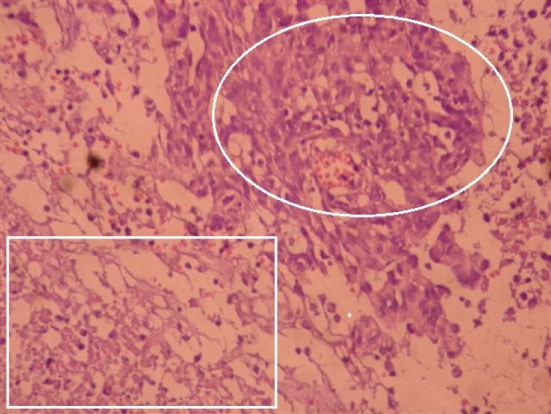
Substantial cellurarity increase and increased mitosis activity in the cells (Field marked with a circle surrounded), wide necrosis and bleeding areas (field marked with a square surrounded)

**Figure 4:**
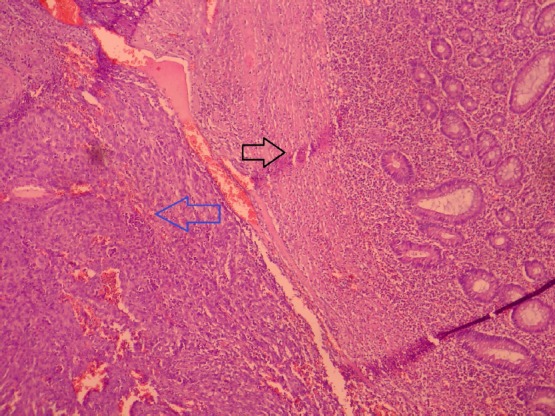
The relationship of tumoral tissue with intestinal mucosa. The area shown by black arrow is intestinal mucosa. The area shown by blue arrow is tumoral area

Uterus couldn’t be observed in transvaginal ultrasound due to previous operation. Right ovary was 22×17 mm in size and normal in appearance. A semisolid appearing cystic mass 47×44 mm in size was observed in the area concordant with left ovarian lodge. Left ovarian border couldn’t be clearly detected. Among tumor markers cancer antigen 125 (Ca125): 21 IU/ml, Ca19-9: 12 IU/ml, carcinoembryonic antigen (CEA): 2 IU/ml, alpha feto protein (AFP): 0.8 IU/ml were in normal range. Enhanced computerized tomography has revealed solid lesion with prominent enhanced material fixation and a necrotic component in the central in left ovarian lodge; the lesion had bilobular appearance and was 66×45 mm in size (Figure 1). In light of these information the patient was operated after taking informed consent from the patient. We entered abdomen by a median incision below the umbilicus under general anesthesia. General evaluation after obtaining abdominal lavage fluid has indicated that omentum was attacking towards left inguinal region. Uterus couldn’t be seen due to previous operation. Right ovary was in normal location but atrophic and in the left ovary lodge a mobile semisolid cystic mass with partially regular borders app. 40×40 mm in size was observed. The mass was attached to ovarian tissue. Ovary was atrophic and in normal appearance. When it’s recognized that existing mass started from serosa of distal ileum a general surgeon was invited for surgery. Mass was removed along with 15 cm intestinal segment and frozen section was done. Macroscopic examination of the mass revealed that it’s adjacent to intestinal segment and nodular with regular outer surface and 7*5*4 cm in size. Section surface was relatively soft in consistency and included occasional yellow coloured regions concordant with necrosis (Figure 2). The result of frozen was reported as gastrointestinal stromal tumor. Exploration showed that there was no metastasis in abdominal cavity. After end-to-end anastomosis, bilateral salpingo oophorectomy and partial omentectomy the operation was terminated. Microscopic examination of the excised tissue revealed that it’s considered generally from proliferating spindle cells showing tumoral features. Prominent increase in cellularity and increase in mitosis activity of cells (grade 2) with accompanying wide necrosis and bleeding areas were observed (Figure 3). In some regions tumoral tissue was attached to intestinal mucosa and partial mucosal ulceration areas were recognized (Figure 4). CD117 (C-KIT) staining by immunohistochemical method performed in an external lab for differential diagnosis revealed diffuse and strong positivity, Ki 67 positivity was 40% and Pan Cytokeratin was negative. The images couldn’t be retrieved from the external lab. Pathologic stage was stage IIIb, there was no complication in the post-op follow up and the patient was referred to medical oncology clinic for maintenance therapy.

## 3. Discussion

GISTs were referred as various sarcomas for years but electron microscopes and immunohistochemical staining have revealed that these start from Cajal cells which are intestinal pacemaker cell (Levy et al., 2003). GISTs may occur as totally myoid, neural or ganglionic plexus phenotype or as a mixture of incompletely differentiated phenotypes (Gold & De Matteo, 2006). GISTs most commonly start from stomach followed by small intestine and in rare case reports in the gynecology literature it was recognized that masses suspected as gynecological lesions originate from small intestine and stomach. In nearly all of the cases in the literature preoperative diagnosis was incorrect. This is due to the false image of GIST in the ultrasound such that it resembles a nonspecific mass with a central hyperechogenic focus resulting from myxoid degeneration. This leads to the confusion of GIST with uterus leiomyosarcoma or ovarian masses that are frequently observed in gynecology practice (Pinto et al., 2007). As it’s true for case report published by Belics et al. (2003), the patient may be operated with a preliminary diagnosis of mature cystic teratoma based on imaging modalities (Belics et al., 2003). Our case was compliant with mature cystic teratoma in enhanced CT examination.

In a series of 191 patients it was observed that incidentally detected GISTs started usually from stomach, colon and rectum. In this series it was determined that GIST cases starting from small intestine have referred for medical help due to various gastrointestinal symptoms (Hassan et al., 2008). However, as it’s true for our case, sometimes the disease may be detected in patients incidentally during a routine examination. In other locations incidental diagnosis may be established by endoscopy or imaging method but as in our case small intestine GIST usually may be detected intraoperatively (Hassan et al., 2008). When tumor diameter increases in GISTs to differentiate it from other tissues and to determine their operability, diagnostic process must include enhanced computerized tomography (CT) and magnetic resonance imaging (MRG) (Sharp, Ansel, & Keel, 2001). In CT imaging small sized tumors may be seen as intramural mass. Large tumors (>5cm) usually grow outside intestines and may move towards pelvic floor by the effect of gravity. Particularly imaging results of GISTs growing in this fashion may be interpreted as originating from ovaries as exemplified in our case. Thus, the most successful differential diagnosis of GISTs from other abdominopelvic masses can be established by description of its relation with adjacent organs and detecting the origin of the disease precisely (Miettinen et al., 2002). But, preoperatively this can’t be always done. Additionally, due to the compromise in blood supply into the tumor, center of the mass could be observed as necrosis and caseification areas in CT. In our case, CT revealed a solid lesion 65×45 mm in size in the left ovarian lodge showing prominent contrast material fixation in enhanced images; the lesion was bilobular and there was a necrotic component in the center.

GISTs starting from small intestine and colon are more aggressive malignancies relative to stomach (Pierie et al., 2001). These tumors metastasize to lymph nodes and most frequently to liver and periton. Prognosis of GISTs with peritoneal and hepatic metastasis is worse. Metastasis to lung and bone occur after metastasis to former tissues (D’Amato et al., 2005). Unfortunately, GIST is not a kind of tumor that prognostic characteristic can be predicted from its pathological features and clinical course. Nevertheless, size, mitotic activity, location of origin and presence of C-KIT mutation are significant in predicting prognosis (Fletcher et al., 2002). Tumor diameter > 5 cm and detection of coagulative necrosis are indicators of bad prognosis. Presence of C-KIT mutation also is mentioned to be related with worse prognosis and recurrence, though disputed (Miettinen, El-Rifai, H. L. Sobin, & Lasota, 2002). In our case size of the tumor was >5 cm and mitotic activity was grade 2. In addition, C-KIT mutation was common and strongly positive. Detection of KIT mutation in pre-operative peripheral blood is possible also in numerous other sarcomas, thus it’s not diagnostic (Taniguchi et al., 1999). In GIST expressing C-KIT (CD 117) exon 11 mutations is detected and in PDGFRA mutation exon 18 mutations is detected (Ernst et al., 1998). However, coexistence of CD 34 and CD 117 positivity in GIST is highly helpful in differential diagnosis. If CD34 is positive CD 117 is also almost always positive (Fletcher et al., 2002). In our case, since the diagnosis was established per-operatively these markers weren’t studied. GIST adenocarcinoma (75%) may be concomitant with lymphoma and carcinoid tumors. 14% of GISTs and 25% of GISTs starting from stomach may co-exist with a second gastrointestinal system malignancy (Miettinen et al., 2002). In GIST genitourinary system cancers are the most frequent co-existing cancers. In a series of 783 patients GUS tumors was 8%, GIS extra tumors was 6% (Pandurengan et al., 2010). In our case, other tissues were evaluated with great attention to look for extra co-existing malignancy, but no other malignancy was detected.

In GIST, main target in surgery is to remove the tumor by protecting pseudo capsule without rupture and with negative microscopic border. To achieve this target, wedge resection or segmental resection 1 cm away from macroscopic border is usually sufficient (DeMatteo et al., 2000). Since lymph mode metastasis is a rare occurrence, there is no lymphadenectomy indication (Demetri et al., 2007). We operated our patient in compliance with these rules and found out that surgical border is negative. In half of the patients whom localized GIST was totally removed by surgery recurrences occur within two years. This end up with mortality in half of the patients within five years (Singer et al., 2002). To prevent recurrence and metastasis surgical therapy alone is not sufficient and other modalities such as radiotherapy and chemotherapy should be added. However, sometimes cases may be highly resistant to these treatment modalities (DeMatteo et al., 2000). Even though effectiveness of thyrosine kinase inhibitors used in chemotherapy such as imatinib, sunitinib, sorafenib, dasatinib, IPI504, oblimersen was shown in patients with tyrosine kinase positivity, adjuvant therapy is still under investigation and may be added to the therapy as neoadjuvant therapy in large tumors (Heinrich & Corless, 2005). In our patient, imatinib therapy was started in medical oncology department after removal of the mass along with an intestinal segment without leaving any tumoral area macroscopically in our gynecology department. In the post-op follow up of our patient there was no recurrence.
